# Electrochemically-Obtained Polysulfonic-Acids Doped Polyaniline Films—A Comparative Study by Electrochemical, Microgravimetric and XPS Methods

**DOI:** 10.3390/polym12051050

**Published:** 2020-05-03

**Authors:** Vladimir Lyutov, Varvara Kabanova, Oxana Gribkova, Alexander Nekrasov, Vessela Tsakova

**Affiliations:** 1 Institute of Physical Chemistry, Bulgarian Academy of Sciences, 1113 Sofia, Bulgaria; vlyutov@ipc.bas.bg; 2 A.N. Frumkin Institute of Physical Chemistry and Electrochemistry, Russian Academy of Sciences, 119071 Moscow, Russia; kabanovavar@gmail.com (V.K.); oxgribkova@gmail.com (O.G.); secp@elchem.ac.ru (A.N.)

**Keywords:** polyaniline, polysulfonic acids, doping, XPS

## Abstract

Polyaniline (PANI) layers are electrochemically obtained in the presence of four polysulfonic acids with different rigidities of the polymer backbone-iso-(and tere-)poly-(4,4′-(2,2′-disulfonic acid)-diphenylene-iso(tere)-phthalamide (i-PASA and t-PASA), polystyrenesulfonic acid (PSSA) and poly(2-acrylamido-2-methyl-1-propanesulfonic acid) (PAMPSA). Combined microgravimetric (EQCM) and electrochemical measurements are carried out in the course of polymerization and repetitive redox switching. It is found that after synthesis PASA-doped PANIs shows good stability with low exchange of mass in the course of voltammetric scans, while PAMPSA-doped PANI contains a large amount of water that gradually becomes expelled in the repetitive redox switching. PANI obtained in the presence of PSSA takes an intermediate position with respect to mass exchanged in the electrochemical redox process. XPS studies are used to obtain data for the extent of doping of the different PANI materials. The results show high doping level (about 0.5) for PASA- and PAMPSA- and lower level (0.32) for PSSA-doped PANI layers. Repeated electrochemical studies carried out with the specimens investigated by XPS after long-term storage in the dry state give evidence for structural rearrangement, perfect recuperation of the initial electrochemical activity and high stability of the electrochemical response.

## 1. Introduction

**Scheme 1:**
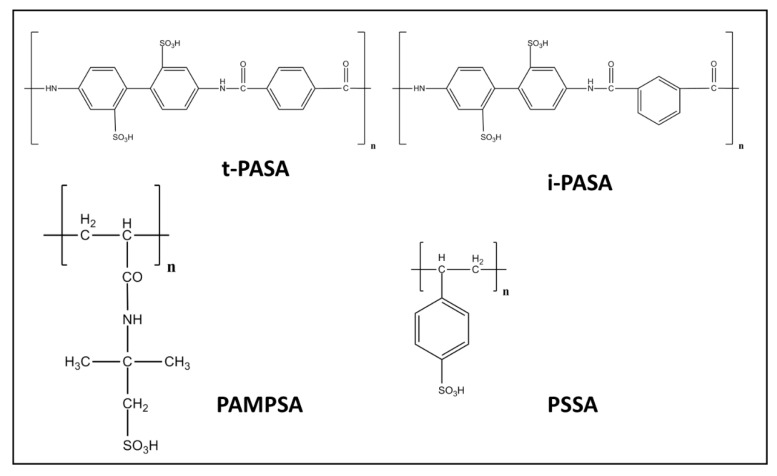
Polysulfonic acids used as dopants for polyaniline (PANI).

Polyaniline (PANI) is one of the most studied conducting polymer materials that provokes continuous interest due to multiple emerging applications [1,2,3]. PANI can be obtained in chemical or electrochemical ways and several synthetic conditions can influence markedly its properties. One of the practical approaches to affect PANI characteristics is the use of different doping ions. Apart from simple inorganic anions (e.g., chloride, sulfate or perchlorate), several sulfonic acids such as camphorsulfonic, dodecylbenzene sulfonic, acrylamidomethylpropane sulfonic or p-toluene sulfonic were also explored with respect to the influence on PANI properties [4,5,6,7,8,9]. Furthermore, electrochemical and/or chemical synthesis of PANI was studied in the presence of polysulfonic acids, e.g., polystyrenesulfonic (PSSA) or poly(2-acrylamido-2-methyl-1-propanesulfonic) (PAMPSA) [10,11,12,13,14,15,16,17,18]. In the recent years a series of papers [19,20,21,22,23,24,25] was devoted to the properties of PANI obtained in the presence of two poly(amidosulfonic acids) that are identical in their chemical composition but differ in the rigidity of their backbone-poly-(4,4′-(2,2′-disulfonic acid)-diphenylene-tere-phthalamide) (t-PASA, rigid backbone) and poly-(4,4′-(2,2′-disulfonic acid)-diphenylene-iso-phthalamide) (i-PASA, semi-rigid backbone) (Scheme 1). On the basis of comparison of electrochemical and spectroelectrochemical properties of these interpolymer PANI films, it was supposed that flexible-chain polyacids can adapt its structure to the rigid conjugated macromolecule of emeraldine form of PANI. In the interpolymer complex with rigid-chain polyacid, such as t-PASA, PANI, in its turn, has to adjust its conformation to match the structure of polyacid. Spectroelectrochemical and electrochemical (cyclic voltammetry) studies of the films showed that the formation of quinoid units at high oxidation levels is retarded in the interpolymer complexes of PANI with rigid- and semi-rigid-chain polysulfonic acids [19,21].

The aim of the present study is to compare PANI layers obtained in the presence of four different polysulfonic acids with respect to kinetics of electrochemical polymerization, electrochemical redox behavior (including type of ionic and solvent fluxes) and extent of doping. To achieve this aim electrochemical, microgravimetric and XPS studies are carried out on the same samples in order to obtain self-consistent information. Furthermore, data for the electrochemical activity of the four types of PANI samples are obtained both directly after synthesis and after XPS studies and long-term storage in the dry state.

XPS studies are commonly used to obtain the extent of doping of PANI materials whereas the electrochemical redox behavior of PANI-coated electrodes is used to evaluate the so-called redox number. The latter is based on data for the polymerization and redox charges and should be also an estimate for doping. PSSA-doped PANIs were studied by means of XPS in several cases [13,14,15,16] but information on their electrochemical behavior obtained with the same materials was not communicated. In some cases [13,16] PANI was obtained chemically but electrochemical studies were not in the focus of interest. In the remaining investigations [14,15] the polymerization was electrochemical but it was carried out in the presence of inorganic ions. In subsequent steps PANI was chemically transformed in the emeraldine base state and thereafter re-doped with PSSA. Additionally, in these cases there was no electrochemical characterization of the PSSA re-doped PANI in terms of electrochemical redox behavior and electrochemical stability upon voltammetric cycling. To our knowledge PAMPSA-, t-PASA- and i-PASA-doped PANIs were not studied yet in the combined way suggested in the present investigation. 

## 2. Materials and Methods

Aniline (Merck Chemicals KGaA, Darmstadt, Germany), PAMPSA (Sigma-Aldrich, now Merck Chemicals KGaA, Darmstadt, Germany), *M*_w_ = 2,000,000, 15% aqueous solution) and sulfuric acid (Merck Chemicals KGaA, Darmstadt, Germany) were used for preparing the synthesis and characterization solutions. PSSNa (Sigma-Aldrich, now Merck Chemicals KGaA, Darmstadt, Germany, *M*_w_ = 10,000,000, 25% aqueous solution) was converted into H^+^-form using ion-exchange column. Both PASA polyacids were obtained from laboratory synthesized i-PASA and t-PASA sodium salts by converting them into the H^+^-forms according to the procedure described in [26]. The molecular weights of the polyacids per monomer repeat unit are: *M*_w_ = 474.32 g/M for t-PASA and i-PASA, *M*_w_ = 184.14 g/M for PSSA (flexible), and *M*_w_ = 207.24 g/M for PAMPSA. The polyacids were diluted with distilled water to obtain 0.05 M PAMPSA or PSSA, 0.025 M i-PASA or t-PASA aqueous solutions. The concentration of i-PASA and t-PASA was reduced twice in order to keep the same aniline per sulfonic group ratio in all solutions. Freshly distilled aniline (0.025 M) was added in the polyacid solutions and they were let for several hours (typically 1–2) on a magnetic stirrer.

Polyaniline was electrodeposited at constant polymerization potential (E = 0.7 vs. Ag/AgCl) in the presence of the different doping ions by interrupting the polymerization at fixed values of the anodic charge, *Q*_p_. PANI specimens with *Q*_p_ = 5 and *Q*_p_ = 25 mC were obtained in each polymerization solution. After polymerization the redox behavior of the PANI coatings was investigated in 0.5 M H_2_SO_4_ by cyclic voltammetry at a scan rate of 50 mV/s. These measurements were repeated on the same specimens after XPS measurements and log-term (12 months) storage in the dry state.

Simultaneous microgravimetric and electrochemical measurements were carried out by means of Metrohm-Autolab BV (Utrecht, the Netherlands) EQCM device coupled to Autolab 302N potentiostat/galvanostat. Gold-coated quartz resonators (6 MHz) with surface area of 0.361 cm^2^ and sensitivity of 4.43 μg/kHz were used as working electrodes for potentiostatic polymerization and voltammetric redox experiments. An Ag/AgCl electrode was used as reference and a gold wire as counter electrode.

X-ray photoelectron spectroscopy (XPS) was carried out by means of an AXIS Supra electron spectrometer (Kratos Analitycal Ltd., Manchester, UK) with base vacuum in the analysis chamber of ~10^−7^ Pa. The spectra were recorded using achromatic Al Kα radiation with a photon energy of 1486.6 eV and a charge neutralization system. The energy scale was calibrated by normalizing the C 1 s line of adsorbed adventitious hydrocarbons to 284.6 eV. The binding energies (BE) were determined with an accuracy of ±0.1 eV. The commercial data-processing software of Kratos Analytical Ltd. (Manchester, UK) was used to calculate the concentrations of the different chemical elements (in atomic %) by normalizing the areas of the photoelectron peaks to their relative sensitivity factors. The deconvolution of the high-resolution element spectra was performed by the ESCApe™ software (Kratos Analytical Ltd., Manchester, UK).

## 3. Results and Discussion

### 3.1. Coupled Electrochemical and Microgravimetric Measurements in the Course of Polymerization

**Figure 1:**
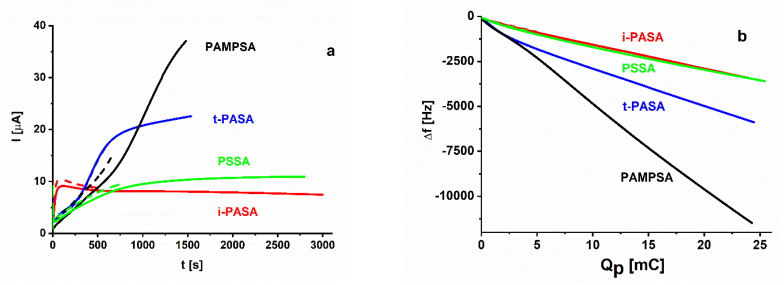
Electrochemical (**a**) and microgravimetric (**b**) measurements in the course of potentiostatic polymerization in the presence of PAMPSA (black), t-PASA (blue), i-PASA (red) and PSSA (green). Full and dashed lines correspond to thick and thin layers, correspondingly.

Current transients obtained in the course of electrochemical polymerization of aniline in the four polymerization solutions and corresponding results from the microgravimetric measurements are presented in Figure 1. 

The type of dopant affects markedly the polymerization kinetics—very fast growth is observed for PAMPSA- and t-PASA-doped PANI whereas i-PASA and PSSA slow down the polymerization. The microgravimetric data show a similar frequency shift with polymerization charge for i-PASA- and PSSA- doped layers whereas t-PASA and especially PAMPSA-doped layers show significant Δf decrease. The most striking effect is observed when comparing growth in the presence of i-PASA and t-PASA which have the same molecular weights and nevertheless result in markedly different frequency response throughout polymerization. Furthermore, at long times PAMPSA-doped PANI shows the largest frequency shift although i-PASA and t-PASA have higher molecular weight per repeat unit than PAMPSA. Apparently, the experimentally obtained frequency shifts do not follow the molecular weights of the doping ions (PSSA<PAMPSA<i-PASA = t-PASA) and thus additional effects such as incorporation of solvent molecules, non-stoichiometric insertion of doping ions (overdoping) and/or dopant-dependent polymerization efficiency should be taken into consideration. 

**Figure 2:**
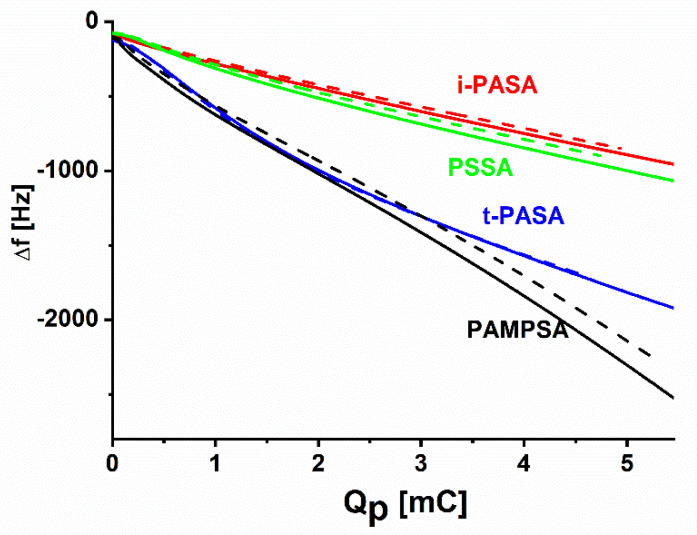
Microgravimetric measurements in the course of polymerization at small polymerization charges in the presence of PAMPSA 9 (black), t-PASA (blue), i-PASA (red) and PSSA (green). Full and dashed lines correspond to thick and thin layers, correspondingly.

For thin PANI layers (see Figure 2) a linear relationship between frequency shift and mass according to Saurbrey equation should be expected to hold. The microgravimetric data allow calculating the apparent molar mass involved in the polymerization process using the formula *M*_app_ = *zF*Δ*m*/*Q*_p_ where Δ*m* is the mass change at passed polymerization charge *Q*_p_, *F* is Faraday’s constant and z is the number of electrons involved in the elementary electrochemical electrodeposition step. The oxidation of the aniline monomer takes two electrons per oxidized unit and additionally there is partial oxidation of the nitrogen atoms in the PANI backbone with maximal value of the number of electrons per monomer equal to 0.5. Table 1 (column 5) shows the data for the M_app_ calculated based on Sauerbrey’s equation for the initial parts of the Δ*f* vs. *Q* dependencies (for *Q* between 0 and 1 mC) by taking the maximal possible extent of oxidation (i.e., *z* = 2.5). The data for t-PASA are evaluated also at a later polymerization stage (for *Q*_p_ between 10 and 11 mC) because of the marked decrease of the slope dΔ*f*/d*Q*_p_ observed for this dopant. The last column in Table 1 shows the theoretical values of the apparent molar mass calculated if assuming fully stoichiometric doping of the PANI charged nitrogen species. For t-PASA and i-PASA it is assumed that both sulfonic groups along the monomer unit are involved in charge compensation. 

The comparison of the data for the apparent molar mass obtained experimentally (column 5) and calculated at stoichiometric insertion of doping ions (column 6) shows close values for the case of i-PASA and thus no over stoichiometric gain of mass is found. In contrast, based on the large experimental *M*_app_ values observed in the PAMPSA and t-PASA(initial stage) cases, overdoping of polysulfonate species and/or significant solvent incorporation should be assumed. PSSA- and t-PASA(advanced stage)-doped PANIs take an intermediate position with about 25% to 33% overweight with respect to the theoretical expectation. The analysis of the microgravimetric data so far performed indicates that the most flexible dopant (PAMPSA) and the rigid t-PASA (in the initial stage) show the largest initial overstoichiometric mass gain, which is persistent for PAMPSA and fades out for t-PASA. For PAMPSA this seems to be an expected effect due to its flexibility and hydrophilicity. The effect of t-PASA should be a different one. Due to its rigidity t-PASA cannot easily adjust to the likewise rigid PANI strands and thus accommodation of both structures seems to take place at a later stage of the building of the bulky net-like polymer structure in which PANI fragments serve as cross-linking chains for several polyacid macromolecules [20,23]. It was shown [27] that t-PASA forms bundle-like supramolecular assemblies which are randomly dispersed in the aqueous solution at the concentrations used. So, presumably, the existence of t-PASA assemblies could facilitate aniline concentrating inside these structures, resulting in some acceleration of PANI synthesis and its overdoping in the initial stages.

### 3.2. Electrochemical Redox Behavior and Stability 

**Figure 3:**
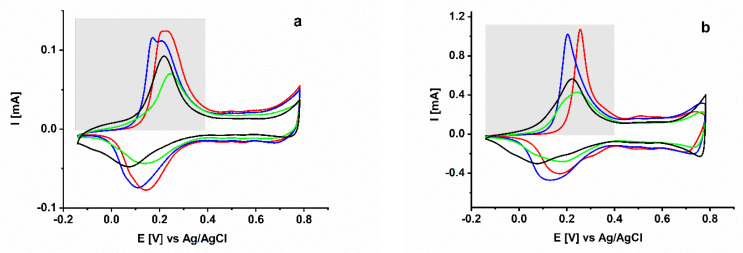
Voltammetric scan measured in 0.5 M H2SO4 at thin (**a**) and thick (**b**) PANI layers obtained in the presence of PAMPSA (black), t-PASA (blue), i-PASA (red) and PSSA (green). Grey rectangles denote the area where integration of the oxidative peaks was carried out to obtain the values of *Q*_ox_.

The electrochemical redox behavior and stability upon several switching between reduced (leucoemeraldine) and semi-oxidized (emeraldine) states was studied by means of cyclic voltammetry in sulfuric acid solution. The voltammetric measurements for thin and thick PANI layers obtained in the presence of the four polyacids are shown in Figure 3. t-PASA- and i-PASA-PANI layers show the largest oxidation peaks with a clearly seen twin-peak structure for the thin films that transforms into very sharp single peaks for the thick ones. Sharp peaks should indicate to a more ordered structure which is more easily oxidized in the t-PASA case. Oxidation/reduction of PAMPSA- and PSSA-doped PANIs are characterized with smaller and much broader peaks with a noticeable shift to more negative potentials in the PAMPSA case. 

The oxidation charge in the voltammetric curves is used to calculate the so-called redox number *y* characterizing the oxidation state of PANI. By equating the number of moles of aniline taking part in the polymerization reaction and in the redox process the following relationship is easily obtained *y = 2Q*_ox_*/(Q*_p_ *− Q*_ox_*)*, where *Q*_ox_ is the oxidation charge under the voltammetric peak obtained in the course of redox cycling. This relationship holds provided 100% polymerization efficiency. The data calculated for thin and thick PANI layers are presented in Table 2. (*Q*_ox_ is calculated in the potential interval −0.14 to 0.4 V for all specimens.) Thick PANI layers show in all cases higher *y* values very probably due to the possibility to obtain longer chains and to achieve advantageous structural re-arrangement with the advancement of growth. Larger *y* values are obtained for t-PASA- and i-PASA- followed by PAMPSA-doped PANIs whereas rather low *y* values characterize both thin and thick PSSA-doped layers. These results correlate with the observed sharp oxidation peaks and the presumed more ordered structure of PANIs obtained with PASA-dopants. In addition, it should be mentioned that, compared to PAMPSA, PASAs-doped PANI films exhibit much more intensive changes of absorbance in the NIR-range during variation of the potential within the range of leucoemeraldine to emeraldine transition [19,25]. 

**Figure 4:**
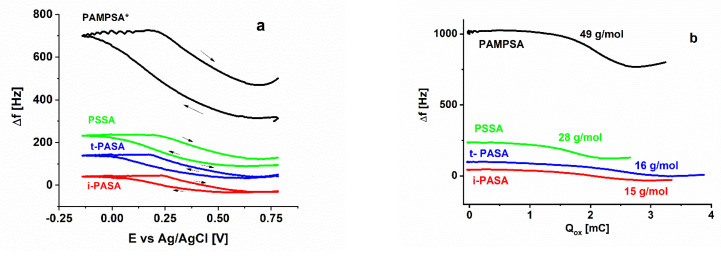
Frequency change in the course of electrochemical redox switching of thick PANI layers obtained in the presence of PAMPSA (black), t-PASA (blue), i-PASA (red) and PSSA (green): (**a**) as function of potential and (**b**) as function of oxidation charge.

Microgravimetric results obtained in the course of single voltammetric cycles measured at the four types of thick PANI layers are presented in Figure 4 in terms of frequency change vs. potential (plot a) and frequency change versus oxidation charge (plot b). It is known that the mass change in the course of redox switching of PANI is due to ionic and/or solvent fluxes necessary to compensate the charge on the PANI backbone and the conformational changes of the polymer structure. As far as polyanionic dopants are incorporated and immobilized in the polymer film in the polymerization stage, hydrogen ions should be expelled to ensure free sulfonate groups for charge compensation. According to Figure 4, for all four types of dopants, the PANI layers gain mass upon oxidation which should be attributed to hydrogen ions release combined with solvent ingress. The apparent molar mass evaluated in the potential range of the leucoemeraldine to emeraldine transition gives M_app_ = 15–16 g per mol charged species for i-PASA and t-PASA, 28 g/mol for PSSA and 49 g/mol for PAMPSA. These values correspond roughly to one (for PASAs), one and half (for PSSA) and three molecules (for PAMPSA) of water involved in the accommodation of one charged species in the PANI backbone. It is apparent that for both t- and i-PASA-doped PANI there is almost no structural relaxation of the polymer structure upon redox switching.

**Figure 5:**
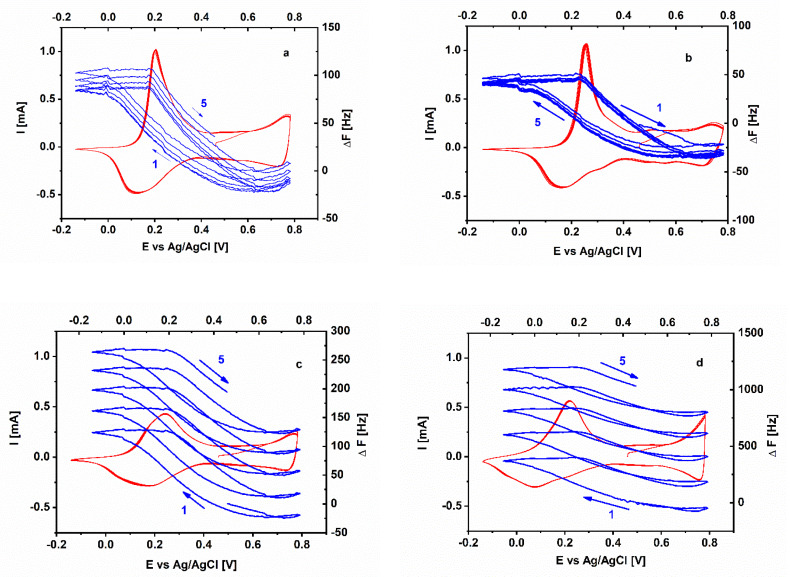
Simultaneous electrochemical (red) and microgravimetric (blue) measurements in the course of five voltammetric scans for (**a**) t-PASA; (**b**) i-PASA; (**c**) PSSA; (**d**) PAMPSA.

The stability of the polyacid-doped PANIs upon repetitive redox switching was checked by measuring several voltammetric scans (Figure 5a–d). These experiments show very good stability of i-PASA-doped PANI from the very first cycle in sulfuric acid after polymerization and almost negligible mass loss for the case of t-PASA. In contrast, PAMPSA-doped PANI shows significant drift to higher frequencies, which means that mass is constantly expelled. Thus, large excess of solvent incorporated during polymerization becomes gradually released upon charging/discharging of the polymer structure. These results are in line with AFM studies [19,23] showing that PAMPSA-doped PANI films, as compared to the films doped with other polyacids, have much looser structure, which may occlude various solution species during the electropolymerization. The loss of mass for PSSA-doped PANI upon continuous cycling is also visible (140 Hz frequency shift in five voltammetric cycles) but markedly smaller in comparison to the PAMPSA case (800 Hz frequency shift in five voltammetric cycles). 

**Figure 6:**
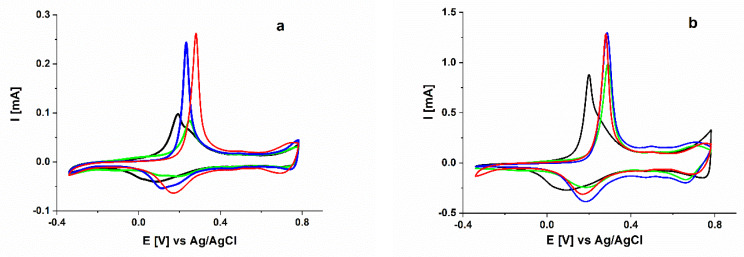
Voltammetric scans measured in 0.5 M H_2_SO_4_ after XPS studies and long-term storage in the dry state of thin (**a**) and thick (**b**) PANI layers obtained in the presence of PAMPSA (black), t-PASA (blue), i-PASA (red) and PSSA (green).

Further electrochemical studies during redox switching (Figure 6) in somewhat extended potential range were completed about 12 months after the specimens were first synthesized, subsequently used for XPS studies and stored in the dry state. Surprisingly, the shape of the cyclic voltammograms for both thin and thick PANIs was found to transform with much sharper leucoemeraldine to emeraldine oxidation peaks observed in all cases. Although the amount of oxidation charge remains practically unaffected, it is obvious that a structural rearrangement has taken place as a result of complete de-hydration (also under vacuum in the XPS chamber). A similar effect could be induced to certain extent by the more negative limits used for the electrochemical reduction. The redox numbers are found to decrease for t-PASA but no significant differences are observed for the remaining three types of PANI layers (Table 2). The microgravimetric data obtained in the course of repetitive voltammetric cycling after the long-term storage in dry state (not shown) give evidence for a perfect reversibility of all types of PANI layers without any mass loss in subsequent scans. The evaluation of the amount of water molecules exchanged in the course of a single voltammetric scan shows similar values for PSSA and PAMPSA to the ones obtained before the storage in the dry state. t-PASA doped PANI, however, is found to exchange by 40% lower amount of solvent which is in-line with the shift of the main oxidation peak to more positive potentials and the expected more compact polymer structure. Thus, after complete de-hydration all four types of PANI layers become easily re-hydrated and preserve the initial electrochemical activity with stable microgravimetric response upon redox switching. 

### 3.3. XPS Studies

**Figure 7:**
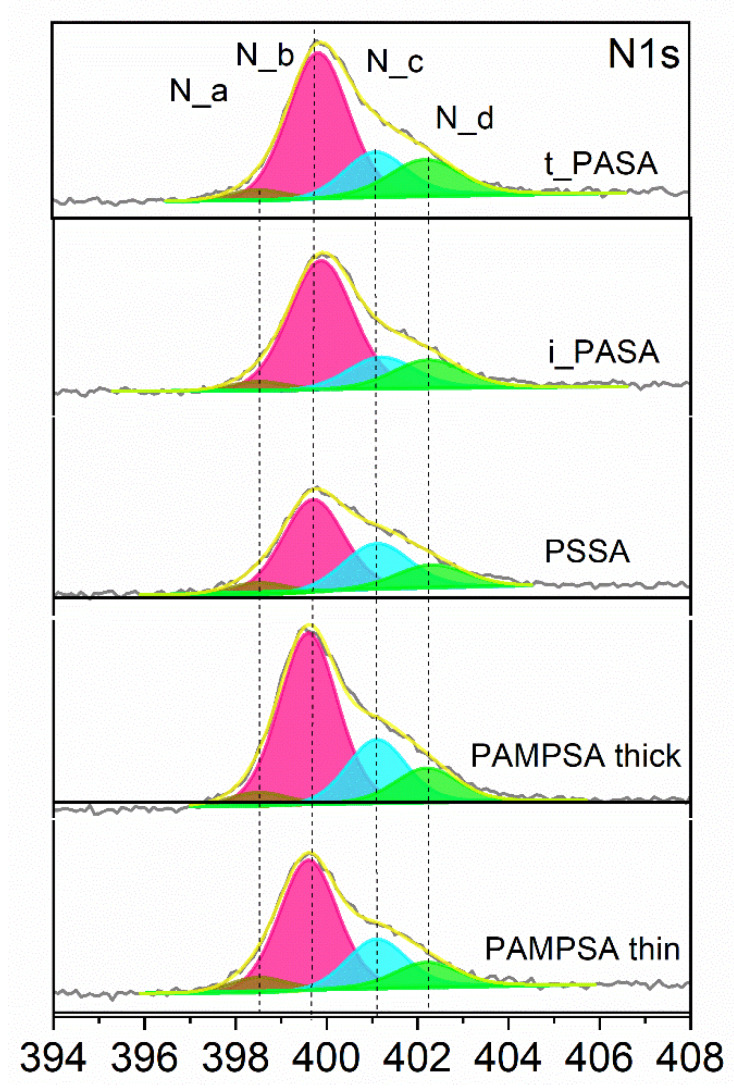
Deconvoluted N 1 s spectra of the different PANI materials.

XPS studies were completed on the PANI specimens used already for coupled electrochemical and microgravimetric measurements. The elemental composition and N 1s deconvoluted spectra (Figure 7) were obtained for five specimens. The nitrogen and sulfur elemental composition of the four types of PANI together with the % contribution of the different N 1s peaks are presented in Table 3. As generally accepted [28,29,30,31] the deconvoluted peaks of the N 1s spectra were attributed to imine (–N = ) (peak a), amine (–NH–) (peak b), protonated amine (–NH^+^) (peak c) and protonated imine ( = NH^+^) (peak d) species with peak positions found at 398.5 eV, 399.7 eV, 401.1 eV and 402.2 eV, respectively. The doping level of PANI is usually estimated by the ratio of the charged nitrogen species to the total nitrogen content. In our case, however, the doping species (PASA and PAMPSA) contain also nitrogen (with stoichiometry S:N = 1:1) which should be excluded from the calculation for the doping level. Thus, for the PASA and PAMPSA cases the total nitrogen atomic content was reduced by extracting the equivalent of the available S content. Therefore except for PSSA the ratio N^(c+d)^/(N–S) was used to estimate the doping level. The doping level for PSSA-doped PANI (N^(c+d)^/N) amounts to 0.32 and is in the range of values (0.30–0.47) obtained for this system in former studies (see Table 4). The data for the doping levels of the PASAs- and PAMPSA-doped PANIs obtained here range between 0.45 and 0.52 (Table 3) which means that practically every second nitrogen atom on the PANI backbone is charged and the theoretical limit corresponding to the emeraldine state is achieved. This somewhat unexpected result should relate to the stabilization of the PANI charged nitrogen species in the interpolymer complexes with the polysulfonic acids as suggested in [19,21]. In fact, in almost all so far explored cases of polysulfonic acid-doped PANI such high doping levels were obtained. In comparison inorganic-ions-doped, as well as mono-sulfonate-doped, PANIs show generally much lower doping levels (Table 4). Furthermore, the XPS data provide the opportunity to estimate the polydopant to PANI ratio, i.e., S_tot_/(N_tot_S_tot_) (for PAMPSA and PASAs) and S_tot_/N_tot_ for PSSA (column (11) in Table 3). By comparing the charged nitrogen species (column (9)) and the amount of doping polyanions (column 11) it becomes evident that except for PSSA all other types of PANI have roughly one to one charged nitrogen to sulfur ratios. Only in the PSSA case there is an excess of polyanionic species that do not participate in the charge compensation.

Finally, one of the specific opportunities in our combined electrochemical and XPS study is the possibility to correlate the doping (based on the amount of charged N species from deconvoluted XPS spectra) and the extent of electrochemical oxidation in the leucomeraldine to emeraldine oxidation transition. The electrochemically obtained redox numbers, y (Table 2) are systematically lower than the ones calculated based on the charged nitrogen species (column 9) or on the protonated amine species alone (column 10). Nevertheless, the electrochemical data for y and the XPS data for the protonated amine species are closer for PASAs-doped PANIs whereas large differences are observed in the PSSA and PAMPSA cases. It can be suggested that the less ordered structures of PSSA- and PAMPSA-doped PANIs is in the origin of overlapping of the NH^+^ and NH = ^+^ oxidation processes which means that the peak due to NH^+^ formation cannot be clearly resolved from the charge of further oxidation. This point remains however open and requires further combined electrochemical and XPS studies.

## 4. Conclusions

The comparative study carried out in the present work shows marked differences in the polymerization behavior of PANI in the presence of two polysulfonic acids with identical chemical composition and different rigidity of the polymer backbone. The rigid t-PASA seems to act as a perfect template to initiate polymerization of aniline without constraints. The net-like structure of the interpolymer complex seems to open enough place for access to the oxidizing aniline units. The process slows down at a later stage and so does the observed overstoichiometric insertion of mass. In comparison, i-PASA decelerates the polymerization from the very beginning due probably to steric hindrances of the more flexible dopant chains. Despite this difference both PASA-doped PANIs show perfect stability upon electrochemical redox switching and very low exchange of mass (presumably one water molecule per PANI oxidative charge). Data obtained from XPS experiment indicate to about 50% doping level of the PASA-doped PANIs.

PAMPSA-doped PANI is characterized by fast polymerization accompanied by massive ingress of solvent molecules that become later gradually expelled in the course of electrochemical redox switching. Apparently, the fast kinetics of PANI growth and random-coil conformation of PAMPSA [23,35] does not allow for structural relaxation in the course of the synthesis. The growth of PANI in the presence of PSSA is strongly limited and this results in both lower extent of doping (32%) and lower incorporation of extra solvent in the polymer structure. These layers undergo re-structuring upon complete de-hydration and show thereafter also a sharp oxidation peak.

The combined electrochemical and XPS studies presented here do not show a clear correlation between electrochemical extent of oxidation (measured by the main PANI oxidation peak) and the doping level of the different PANI layers. The reason should be sought in the fact that the oxidation state of PANI, corresponding to the XPS measurements, does not necessarily coincide with the state obtained right after the leucoemeraldine to emeraldine electrochemical oxidation peak. This issue should be further investigated by taking into account also the possibility for a lower (than 100%) efficiency of the polymerization process that may affect the *y* values. 

Finally, it is worth emphasizing that complete de-hydration of the polyacid-doped PANI layers results in re-ordering of the interpolymer complex structure of all four types of PANI materials. Emerging of sharp voltammetric oxidation peaks with perfect reversibility and stability is observed together with reversible dopant-dependent exchange of mass (ingress and egress of water molecules) upon voltammetric redox switching. All four types of PANI layers are found to preserve their electroactivity under long-term storage in the dry state. 

## Figures and Tables

**Table 1:** Apparent molar mass of the species deposited in the course of polymerization.

Dopant	Molar Mass of Dopant ^i^ g/mol	Δ*f* at *Q*_poly_ = 1 mC/ kHz	Mass Change/ µg	*M*_app_ Experiment/ g/mol	*M*Stoichiomet. Doping/ g/mol
(1)	(2)	(3)	(4)	(5)	(6)
t-PASA	474	0.48	2.129	514	209.5
		0.26 ^ii^	1.152 ^ii^	278 ^ii^	209.5
i-PASA	474	0.19	0.849	205	209.5
PSSA	184	0.21	0.948	229	183.0
PAMPSA	207	0.46	2.053	495	194.5

^i^ per repeat monomer unit; ^ii^ frequency change in the *Q*_poly_ interval 10 to 11 mC (advanced stage).

**Table 2:** Redox numbers obtained from electrochemical data after synthesis and after long-term storage in the dry state.

Dopant	t-PASA		i-PASA		PSSA		PAMPSA	
Type of Film	Thin	Thick	Thin	Thick	Thin	Thick	Thin	Thick
After synthesis	0.20	0.24	0.18	0.20	0.12	0.15	0.13	0.20
After storage	0.17	0.20	0.17	n. a.	0.12	0.15	0.14	0.20

**Table 3:** Data for the atomic content of nitrogen and sulfur and relative rations of N obtained from XPS studies and for the electrochemical redox number.

N	Dopant	N_tot_ at %	S_tot_ at %	N(a) 398.5 eV %	N(b) 399.7 ^i^ eV %	N(c) 401.1 eV %	N(d) 402.2 eV %	N(c+d)/ (N_tot_-S_tot_)	Nc/ (N_tot_-S_tot_)	S_tot_/ (N_tot_-S_tot_)
	(2)	(3)	(4)	(5)	(6)	(7)	(8)	(9)	(10)	(11)
1	t-PASA	8.90	2.85	4.5	60.7	18.2	16.6	0.51	0.27	0.47
2	i-PASA	8.98	2.85	4.37	64.79	15.93	14.90	0.45	0.23	0.46
3	PSSA	7.24	2.80	6.26	52.26	27.40	14.08	0.32 ^ii^	0. 27 ^ii^	0.39 ^ii^
4	PAMPSA-thick	10.61	3.59	4.8	60.7	22.1	12.4	0.52	0.33	0.51
5	PAMPSA-thin	10.17	3.45	7.07	59.8	21.85	11.28	0.50	0.33	0.51
				% from N_total_						

^i^ Exact peak positions for: t-PASA, 399.8 eV, i-PASA, 399.9 eV, PSSA and PAMPSA 399.7 eV; ^ii^ The ratios are taken as N(c+d)/N, N(c)/N, and S_tot_/N_tot_, respectively, as PSSA does not contain nitrogen.

**Table 4:** Data for the deconvolution of the N spectra and the extent of doping of PANIs synthesized in the presence of different doping anions.

Dopant, Synthetic Method	Deconvoluted N 1 s Peak Peak Positions (eV)				XPS Ref. Line	Doping %	Ref.
	–N = (a)	–NH– (b)	–NH^+^ (c)	= NH^+^ (d)			
PSSA, chemical synthesis	398.1	399.3	400.1	401.3	n.a.	30–38	[13]
PSSA, elchem. synthesis in H_2_SO_4_ chemical reduction to EB; redoped	398.8	399.6	400.7	401.8	n.a.	32	[12]
PSSA or PAA, elchem. synthesis in H_2_SO_4_, chemical reduction to EB, redoped	398.8	399.6	400.7	401.8	n.a.	47	[14]
PSSA - elchem. synthesis in H_2_SO_4_ chemical reduction to EB, redoped	398.7	399.6	400.5	401.4	n.a	35–40	[15]
PSSA, PAMPSA chemical synthesis of ES, deprotonation to EB	398.5	399.5	401.1	402.2	284.6 C 1s	43	[16]
dodecylbenzene sulfonic acid, chemical synthesis	398.2	399.6	400.8	402.0	284.6 C 1s	46	[7]
p-toluenesulfonate, camphorsulfonate, chemical synthesis, doped EB	398.5	399.5	401.1	402.2	284.6 C1s	22.5 29.2	[9]
Cl^−^, electrochem. synthesis	No peak	399.2	401.1	403.1	284.8 C 1s	25.4	[32]
Cl^−^, electrochem. synthesis	398.5	399.6	401.1	402.6	284.5 C 1s	20–25 thin 12–13 thick	[33]
dodecylsulfate, chem. synthesis	398.6	399.2	above 400			51.2	[34]
